# Utility of Goldmann applanation tonometry for monitoring intraocular pressure in glaucoma patients with a history of laser refractory surgery

**DOI:** 10.1371/journal.pone.0192344

**Published:** 2018-02-05

**Authors:** Sang Yeop Lee, Hyoung Won Bae, Hee Jung Kwon, Gong Je Seong, Chan Yun Kim

**Affiliations:** 1 Department of Ophthalmology, Severance Hospital, Institute of Vision Research, Yonsei University College of Medicine, Seoul, Korea; 2 Department of Ophthalmology, CHA Bundang Medical Center, CHA University, Seongnam, Republic of Korea; Xiamen University, CHINA

## Abstract

The utility of Goldmann applanation tonometry (GAT) for monitoring intraocular pressure (IOP) in open-angle glaucoma (OAG) patients with a history of laser refractive surgery was investigated by comparing IOP fluctuations measured using GAT and dynamic contour tonometry (DCT) on the same day. In this retrospective study, patients were divided into one of two subgroups according to IOP fluctuation values using GAT: 43 eyes in the low IOP fluctuation group (LIFG [GAT fluctuation ≤1.7 mmHg]); and 55 eyes in the high IOP fluctuation group (HIFG [GAT fluctuation >1.7 mmHg]). IOP fluctuation was defined as the standard deviation of all IOP values during follow-up. IOP parameters using GAT were compared with those of DCT. Correlation analyses were performed among IOP parameters, and between IOP fluctuation and associated factors including central corneal thickness, corneal curvature, and axial length. All IOP parameters demonstrated significantly high values in the HIFG compared with those in the LIFG. Mean and peak IOP using DCT were significantly higher than those using GAT in both groups. However, there were no significant differences in IOP fluctuation and reduction using both tonometry methods in the HIFG (*p* = 0.946 and *p* = 0.986, respectively). Bland-Altman analysis revealed similar fluctuations using GAT and DCT. In multivariate analyses, there was a significant correlation between fluctuations using GAT and DCT in the HIFG (*p* = 0.043). These results suggest that IOP monitoring using GAT is a reliable method of monitoring IOP change in glaucoma patients with a history of laser refractive surgery, especially those exhibiting high IOP fluctuation. Nevertheless, several factors, including central corneal thickness, corneal curvature, and axial length, should be considered when using GAT for IOP monitoring.

## Introduction

Increased intraocular pressure (IOP) is a well-known risk factor for the development and progression of glaucoma [1–4], as are fluctuations in IOP [5]. Therefore, IOP measurement is a basic and essential examination for treatment and follow-up in patients with glaucoma. Although various devices have been developed for IOP measurement, Goldmann applanation tonometry (GAT) is regarded as the gold standard. However, one limitation of IOP measurement using GAT is that accuracy is influenced by biomechanical properties of the cornea, including central corneal thickness (CCT) and corneal curvature (CC) [6, 7].

Laser refractive surgery (LRS), such as laser in situ keratomileusis (LASIK) or laser epithelial keratomileusis (LASEK), alters corneal biomechanical properties, which in turn can lead to inaccuracies in IOP measured using GAT [8–10]. As a way to overcome this limitation, the Pascal dynamic contour tonometer (DCT; SMT Swiss Microtechnology AG, Port, Switzerland) was developed. The concave tip shape of the DCT device minimizes alterations in corneal shape. It is known that DCT measurements closely reflect actual IOP [11]. Several previous studies have demonstrated that changes in IOP before and after LRS are significantly smaller using DCT than GAT [9, 12, 13]. Given that myopia is a risk factor for the development of glaucoma, measuring IOP and monitoring IOP changes are crucial in individuals who have undergone LRS for myopia correction. Glaucoma can be diagnosed using devices for the evaluation of structural or functional glaucomatous retinal nerve fiber layer (RNFL) damage. However, monitoring IOP changes should be performed using tonometry. Although monitoring IOP change is important for glaucoma patients who have undergone LRS, previous studies have primarily focused on the accuracy of IOP values before and after LRS according to different types of tonometry, including DCT.

In this study, we investigated the value of IOP measurement and monitoring using GAT in patients with open-angle glaucoma (OAG) who underwent LRS by comparing IOP parameters, including IOP fluctuation measured using both GAT and DCT on the same day. In addition, we also investigated correlations between ocular parameters (CCT, CC, and axial length) and IOP fluctuation.

## Materials and methods

This retrospective study was conducted at the Department of Ophthalmology, Severance Hospital, Yonsei University School of Medicine (Seoul, Korea), with approval from the Institutional Review Board of Severance Hospital, Yonsei University (2015.9.25). All research adhered to the tenets of the Declaration of Helsinki, and informed written consent was obtained from all subjects. The medical records of patients who visited the Glaucoma Clinic of the Department of Ophthalmology at Severance Hospital, Yonsei University School of Medicine, between January 2005 and June 2015 were reviewed. Patients who underwent LASIK or LASEK at least 3 years before initial visit to the clinic and diagnosed with glaucoma exhibiting an open angle in the clinic using anti-glaucoma topical medication were selected. The follow-up period after initial diagnosis of glaucoma should be > 12 months. IOP was measured using GAT and DCT separately on the same day. At least 6 IOP measurements using GAT and DCT and regular clinic visits were required for inclusion. Other inclusion criteria were as follows: each examination to evaluate glaucoma was performed > 3 times; best-corrected visual acuity (BCVA) was > 20/30; no medical history of systemic disease; and no history of anti-glaucoma medication before the initial clinic visit. Glaucoma patients who underwent ophthalmic surgery, such as cataract surgery, vitrectomy, or glaucoma surgery, including laser trabeculoplasty, were excluded. Additionally, patients who discontinued using anti-glaucoma medication during the follow-up period(s), those who used steroid or non-steroidal anti-inflammatory eye drops, or those with a history of using these eye drops within 3 months of the last follow-up, were also excluded.

The ophthalmic examinations included the following measurements: Snellen BCVA; refractive spherical equivalent; CCT using an ultrasonic pachymeter (DGH-1000; DGH Technology, Inc., Frazer, PA, USA); CC; and AXL using the IOL Master (Carl Zeiss Meditec AG, Jena, Germany). For diagnosis and follow-up of glaucoma, a +90 diopter lens, red-free photograph (VISUCAM 200, Carl Zeiss Meditec AG, Jena, Germany), and Cirrus HD Optical coherence tomography (Carl Zeiss Meditec, Inc., Dublin, CA, USA) were used for the evaluation of disc morphology changes and RNFL defects. Visual field measurements, using the Humphrey Visual Field analyzer (24–2 Swedish Interactive Threshold Algorithm, Carl Zeiss Meditec, Inc., Dublin, CA), were also performed. Medical history regarding changes in anti-glaucoma medication was also reviewed.

### Diagnosis of glaucoma

Diagnosis of glaucoma was re-evaluated on the basis of characteristics of glaucomatous optic disc change and RNFL and visual field defects. Visual field defects had to satisfy at least two of the Anderson criteria [14]. Two glaucoma specialists (S.Y.L and H.Y.B) reviewed the medical records of glaucoma patients. If there was disagreement between these specialists, the medical record was reviewed by a third glaucoma specialist (C.Y.K), who decided whether the diagnosis was correct or incorrect. If both eyes met the study criteria, the study eye was randomly selected.

### IOP measurement and IOP parameters

IOP measurements using GAT and DCT were conducted according to standard procedure at the authors’ institution. Three consecutive measurements were obtained. If IOP values had differences > 3 mmHg, additional measurements were performed. Finally, the mean value of three measurements not showing large differences was calculated and recorded. DCT values with a quality of 1 or 2 were used for calculations. If there was no description of quality in the medical record, the DCT value was not used. Four IOP parameters were used in this study. IOP values measured using each tonometry method in the medical record were averaged (mean IOP) and the maximum IOP value (peak IOP) was extracted. To compare the extent of IOP reduction attributed to anti-glaucoma medication, the difference between peak IOP and mean IOP was calculated (IOP reduction). For the fluctuation of IOP, standard deviation of all IOP values during follow-up periods was calculated (IOP fluctuation). IOP fluctuation was regarded to be a significant factor reflecting IOP change for follow-up period. ΔIOP fluctuation was defined as the difference between DCT fluctuation and GAT fluctuation.

In the present study, subjects were divided into one of two groups according to IOP fluctuation measured using GAT: the relatively low IOP fluctuation group (LIFG); and the relatively high IOP fluctuation group (HIFG). Although there have been several studies investigating long-term IOP fluctuation [5], standard values of normal fluctuation have not been established. Hong et al [15] divided IOP fluctuation―defined as the SD of GAT values―into high- and low-fluctuation groups separated by a pressure of 2.0 mmHg. Considering the underestimation of GAT values affected by CCT, it was decided to set the IOP fluctuation standard value at 1.7 mmHg, which was > 15% of the mean GAT value of all patients in the present study (1.67 mmHg) and < 2.0 mmHg (referenced from Hong et al [15]).

### Statistical analysis

Statistical software (SAS version 9.2, SAS Inc., Cary, NC, USA) was used to perform all statistical analyses. Independent two-sample t tests and chi-squared tests were used to compare differences in continuous parameters and categorical parameters between the groups, respectively. Paired t tests were performed to compare within-group values of the IOP parameters. Pearson’s correlation coefficients and multiple regression were used for correlation analyses. Statistical significance was defined as *p* < 0.05.

## Results

A total of 387 eyes of 194 OAG patients with a history of LASIK or LASEK were selected after review of medical records. Among these patients, 98 eyes of 98 patients who met the inclusion and exclusion criteria were included in the present study. Patients were divided into one of two subgroups according to the IOP fluctuation value obtained using GAT. Table 1 summarizes demographic information and comparisons between LIFG and HIFG. Except for IOP parameters (mean IOP, peak IOP, IOP fluctuation, IOP reduction, and ΔIOP fluctuation), there were no significant differences in other parameters. In the between-group comparisons, all IOP parameters of the HIFG were higher than those of the LIFG, with the exception of ΔIOP fluctuation. Mean and peak IOP measured using DCT (mean DCT and peak DCT) were significantly higher than mean and peak IOP measured using GAT (mean GAT and peak GAT) in each group. IOP fluctuation and reduction measured using DCT (DCT fluctuation and DCT reduction) were significantly higher than those measured using GAT (GAT fluctuation and GAT reduction) in the LIFG (*p* = 0.003, *p* = 0.004, respectively). However, there were no significant differences in IOP fluctuation and reduction between the two types of tonometry in the HIFG (*p* = 0.946, *p* = 0.986, respectively).

**Table 1:** Comparison of demographic and clinical features between the low^a^ and high^b^ intraocular pressure fluctuation groups.

	All group (Mean±SD or ratio)	LIFG^a^ (Mean±SD or ratio)	HIFG^b^ (Mean±SD or ratio)	p-value
**Eyes**	98	43	55	
**Age (years)**	40.78±7.03	41.14±7.61	40.49±6.59	0.653
**Sex (M:F)**	40:58	17:26	23:32	0.839*
**Follow-up (months)**	50.57±42.38	54.56±31.15	47.45±34.19	0.413
**Years after operation**	11.53±4.65	11.51±5.3	11.55±4.13	0.972
**Operation type (LASIK: LASEK)**	56:42	24:19	32:23	0.84*
**Anti-glaucoma medication change (No: Yes)**	48:50	25:18	23:32	0.154*
**Number of medication change**	1.28±1.84	0.88±1.16	1.58±2.19	0.06
**CCT (μm)**	465.9±47.3	466.2±43.1	465.70±50.6	0.96
**AXL (mm)**	27±1.76	26.80±1.58	27.15±1.89	0.386
**CC (D)**	38.02±2.4	38.27±2.72	37.82±2.14	0.422
**SE (D)**	-1.58±1.8	-1.29±1.19	-1.82±2.16	0.153
**MD (dB)**	-5.02±4.51	-4.86±3.85	-5.15±5	0.753
**PSD (dB)**	5.19±4.08	5.16±4.03	5.22±4.15	0.937
**VFI**	88.71±12.26	90.09±11.09	87.62±13.11	0.328
**IOP parameters**				
**Mean (DCT-GAT) (mmHg)**	5.52±1.57	5.4±1.25	5.6±1.78	0.518
**(DCT-GAT) fluctuation (mmHg)**	1.76±0.94	1.53±0.93	1.93±0.91	0.038
**GAT mean (mmHg)**	11.15±2.02	10.66±2.0	11.54±1.97	0.031
**DCT mean (mmHg)**	16.68±2	16.03±1.82	17.18±2.01	0.004
p-value**		<0.001	<0.001	
**GAT peak (mmHg)**	13.73±2.88	12.12±2.25	15±2.7	<0.001
**DCT peak (mmHg)**	19.53±2.89	18.11±2.00	20.65±3.00	<0.001
p-value**		<0.001	<0.001	
**GAT fluctuation (mmHg)**	1.77±0.68	1.16±0.36	2.24±0.44	<0.001
**DCT fluctuation (mmHg)**	1.93±0.88	1.53±0.77	2.23±0.84	<0.001
p-value**		0.003	0.946	
**GAT reduction (mmHg)**	2.58±1.62	1.46±0.61	3.46±1.62	<0.001
**DCT reduction (mmHg)**	2.86±1.73	2.08±1.27	3.47±1.81	<0.001
p-value**		0.004	0.986	
**ΔIOP fluctuation (mmHg)** **(DCT fluctuation- GAT fluctuation)**		0.37±0.78	-0.01±0.8	0.019

* Chi-square test

** Paired t-test

^a^ Low intraocular pressure fluctuation group (LIFG; Goldmann applanation tonometry [GAT] fluctuation ≤1.7 mmHg)

^b^ High intraocular pressure fluctuation group (HIFG; GAT fluctuation >1.7 mmHg)

CCT = central corneal thickness; AXL = axial length; CC = corneal curvature; SE = spherical equivalent; MD = mean deviation; PSD = pattern standard deviation; VFI = visual field index; DCT = Dynamic contour tonometer

### Agreement between IOP fluctuation values measured using the two different tonometry methods

**Fig 1:**
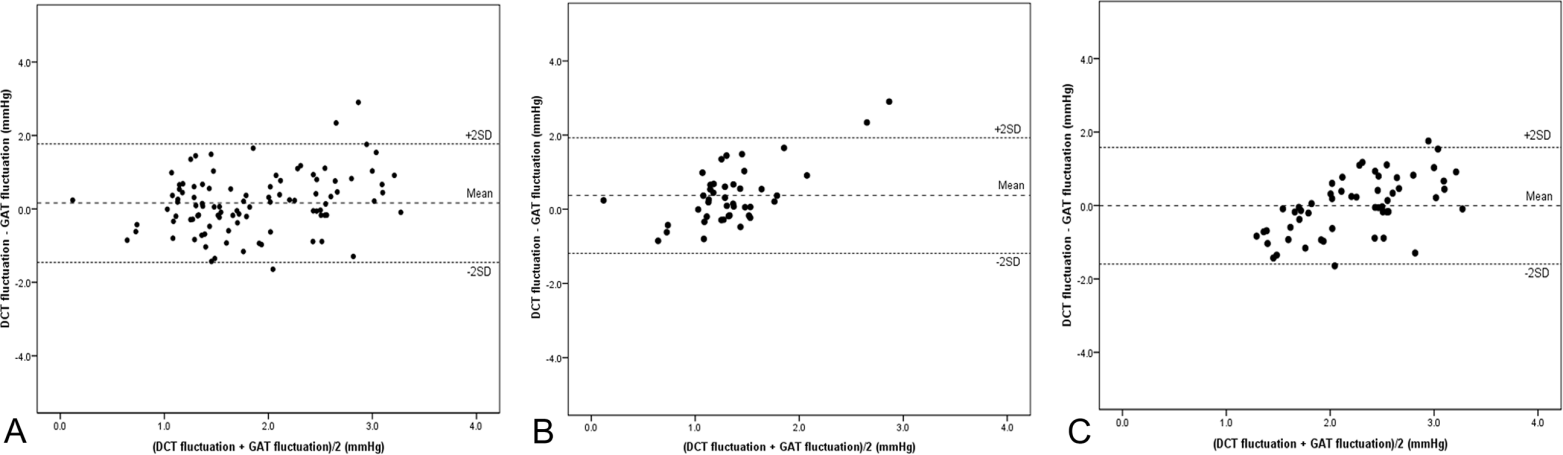
Bland-Altman plots of Goldmann applanation tonometry (GAT) fluctuation and dynamic contour tonometry (DCT) fluctuation in all patients (A); the low intraocular pressure (IOP) fluctuation group (B); and the high IOP fluctuation group (C).

Bland-Altman plots of IOP fluctuations measured using DCT and GAT are shown in Fig 1. The agreement between the two values of IOP fluctuation was verified overall and in each group. The mean values of the difference between the two values of IOP fluctuation were 0.16 mmHg, 0.37 mmHg in LIFG and HIFG, respectively, and -0.001 mmHg overall. Most values were within ± 2 SD of the difference between two values of IOP fluctuation in each group.

### Correlation analyses of IOP parameters

Table 2 summarizes the results of correlation analyses among IOP parameters. Correlation analyses of mean IOP, peak IOP, IOP fluctuation, and IOP reduction values between the two different tonometry methods were conducted in each group. Additionally, correlations between IOP fluctuation and mean IOP values were also analyzed. Correlations between mean GAT and mean DCT were significant in both groups. Peak GAT and peak DCT also demonstrated significant correlations in both groups. Correlations between GAT fluctuation and DCT fluctuation, and between GAT and DCT reduction were not significant in the LIFG (r = 0.211; *p* = 0.174 and r = 0.141, *p* = 0.357, respectively). GAT fluctuation demonstrated significant correlations with both mean GAT and mean DCT in the LIFG (r = 0.475, *p* = 0.001 and r = 0.376, *p* = 0.013, respectively). Additionally, DCT fluctuation showed significant correlations with both mean GAT and mean DCT in the HIFG (r = 0.273, *p* = 0.044 and r = 0.352, *p* = 0.008, respectively). However, no significant correlations were detected between GAT fluctuation and mean IOP in the HIFG, and between DCT fluctuation and mean IOP in the LIFG.

**Table 2:** Correlations between intraocular pressure parameters in each group according to Pearson’s correlation coefficients.

	All groups		LIFG		HIFG	
	r	p-value	r	p-value	r	p-value
**Mean GAT: mean DCT**	0.766	<0.001	0.8	<0.001	0.720	<0.001
**Peak GAT: peak DCT**	0.676	<0.001	0.645	<0.001	0.567	<0.001
**GAT fluctuation: DCT fluctuation**	0.485	<0.001	0.211	0.174	0.354	0.008
**GAT reduction: DCT reduction**	0.505	<0.001	0.141	0.367	0.416	0.002
**GAT fluctuation: mean (DCT-GAT)**	0.102	0.329	-0.242	0.133	0.221	0.109
**GAT fluctuation: (DCT-GAT) fluctuation**	0.310	0.002	0.105	0.502	0.317	0.02
**GAT fluctuation: mean GAT**	0.353	<0.001	0.475	0.001	0.2	0.142
**GAT fluctuation: mean DCT**	0.401	<0.001	0.376	0.013	0.256	0.059
**DCT fluctuation: mean (DCT-GAT)**	0.142	0.172	0.110	0.499	0.137	0.323
**DCT fluctuation: (DCT-GAT) fluctuation**	0.591	<0.001	0.818	<0.001	0.388	0.004
**DCT fluctuation: mean GAT**	0.258	0.01	0.079	0.613	0.273	0.044
**DCT fluctuation: mean DCT**	0.296	0.003	-0.018	0.906	0.352	0.008
**(DCT-GAT) fluctuation: mean GAT**	0.138	0.185	-0.135	0.407	0.255	0.063
**(DCT-GAT) fluctuation: mean DCT**	0.016	0.88	-0.220	0.173	0.067	0.63

LIFG = low intraocular pressure fluctuation group; HIFG = high intraocular pressure fluctuation group; GAT = Goldmann applanation tonometry; DCT = Dynamic contour tonometer

### Correlation analyses of IOP fluctuation and ocular parameters

In each group, correlation analyses between IOP fluctuation and ocular parameters, including CCT, AXL, and CC, were performed (Table 3). GAT fluctuation showed significant correlation with these three ocular factors in the HIFG. In contrast, however, there were no significant correlations between GAT fluctuation and these ocular parameters in the LIFG. AXL was the sole ocular parameter significantly correlated with DCT fluctuation in the LIFG. No significant correlation was detected between DCT fluctuation and these three ocular parameters in the HIFG.

**Table 3:** Correlations between intraocular pressure fluctuation and ocular parameters in each group according to Pearson’s correlation coefficients.

	Mean (DCT-GAT)		(DCT-GAT) fluctuation		GAT fluctuation		DCT fluctuation	
	r	p-value	r	p-value	r	p-value	r	p-value
**LIFG**								
**CCT (μm)**	-0.102	0.542	-0.135	0.417	0.189	0.237	-0.157	0.326
**AXL (mm)**	0.313	0.093	0.540	0.002	0.071	0.696	0.411	0.017
**CC (D)**	-0.282	0.125	-0.484	0.006	0.045	0.802	-0.311	0.073
**HIFG**								
**CCT (μm)**	-0.324	0.018	0.015	0.913	-0.386	0.004	-0.182	0.187
**AXL (mm)**	0.191	0.220	0.068	0.666	0.326	0.031	0.179	0.245
**CC (D)**	-0.189	0.225	-0.028	0.861	-0.409	0.006	-0.197	0.199

LIFG = low intraocular pressure fluctuation group; HIFG = high intraocular pressure fluctuation group; CCT = central corneal thickness; AXL = axial length; CC = corneal curvature; GAT = Goldmann applanation tonometry; DCT = Dynamic contour tonometer

### Multivariate analyses of IOP fluctuation and related variables

In multivariate analyses, age, sex, operation type (i.e., LASEK vs LASIK), CCT, AXL, CC, mean GAT and mean DCT were included as possible factors correlated with GAT or DCT fluctuation. In analyses between GAT fluctuation and other factors, DCT fluctuation was also used as a correlated factor and vice versa. Table 4 summarizes the results of the multivariate analyses investigating GAT fluctuation and related factors. There was no correlated factor in the LIFG. In the HIFG, CCT and DCT fluctuation were significantly correlated with GAT fluctuation (*p* = 0.013 and *p* = 0.043, respectively). Table 5 summarizes the results of multivariate analyses between DCT fluctuation and related factors. Among 9 factors, only GAT fluctuation was significantly correlated with DCT fluctuation in the HIFG (*p* = 0.043). There was no factor correlated with DCT fluctuation in the LIFG.

**Table 4:** Multivariate analyses between Goldmann applanation tonometry fluctuation and related variables in each group.

	LIFG		HIFG	
	β (95% CI)	p-value	β (95% CI)	p-value
**Age (years)**	-0.018(-0.039–0.004)	0.099	-0.014(-0.04–0.12)	0.280
**Sex**				
**Male**	Reference		Reference	
**Female**	-0.227(-0.549–0.096)	0.159	-0.191(-0.434–0.052)	0.119
**Operation Type**				
**LASIK**	Reference		Reference	
**LASEK**	-0.172(-0.527–0.183)	0.326	-0.144(-0.442–0.154)	0.333
**CCT (μm)**	-0.002(-0.006–0.003)	0.397	-0.005(-0.008- -0.001)	0.013
**AXL (mm)**	0.008(-0.177–0.193)	0.925	0.001(-0.088–0.09)	0.978
**CC (D)**	0.048(-0.069–0.166)	0.401	0.008(-0.079–0.096)	0.850
**Mean GAT (mmHg)**	0.11(-0.002–0.221)	0.054	0.02(-0.072–0.112)	0.655
**Mean DCT (mmHg)**	0.027(-0.101–0.155)	0.667	0.006(-0.078–0.09)	0.887
**DCT fluctuation (mmHg)**	0.102(-0.064–0.269)	0.215	0.158(0.07–0.32)	0.043

CI = confidence interval; LIFG = low intraocular pressure fluctuation group; HIFG = high intraocular pressure fluctuation group; LASIK = laser in situ keratomileusis; LASEK = laser epithelial keratomileusis CCT = Central corneal thickness; AXL = axial length; CC = corneal curvature; GAT = Goldmann applanation tonometry; DCT = Dynamic contour tonometer

**Table 5:** Multivariate analyses between dynamic contour tonometer fluctuation and related variables in each group.

	LIFG			HIFG	
	β (95% CI)	p-value	β (95% CI)		p-value
**Age (years)**	0.012 (-0.046–0.071)	0.661		-0.028 (-0.082–0.025)	0.290
**Sex**					
**Male**	Reference			Reference	
**Female**	0.331 (-0.523–1.185)	0.43		0.047 (-0.475–0.568)	0.857
**Operation Type**					
**LASIK**	Reference			Reference	
**LASEK**	0.067 (-0.863–0.997)	0.883		-0.093 (-0.717–0.531)	0.763
**CCT (μm)**	0.002 (-0.01–0.014)	0.752		0.002 (-0.006–0.01)	0.564
**AXL (mm)**	0.178 (-0.29–0.646)	0.438		0.044 (-0.139–0.227)	0.625
**CC (D)**	-0.082 (-0.385–0.221)	0.579		0.054 (-0.126–0.234)	0.548
**Mean GAT (mmHg)**	0.094 (-0.216–0.403)	0.536		0.028 (-0.163–0.218)	0.768
**Mean DCT (mmHg)**	-0.163 (-0.484–0.158)	0.304		0.117 (-0.051–0.286)	0.166
**GAT fluctuation (mmHg)**	0.673 (-0.421–1.766)	0.215		0.674 (0.39–1.365)	0.043

CI = confidence interval; LIFG = low IOP fluctuation group; HIFG = high IOP fluctuation group; CCT = central corneal thickness; AXL = axial length; CC = corneal curvature; GAT = Goldmann applanation tonometry; DCT = Dynamic contour tonometer; LASIK = laser in situ keratomileusis; LASEK = laser epithelial keratomileusis

## Discussion

IOP fluctuation is divided into long- and short-term fluctuations [5]. Although there is no definitional or methodological consensus for these two types of IOP fluctuation, many studies have demonstrated significant correlation between IOP fluctuation and glaucoma [15–19]. Therefore, for patients who have undergone LRS, both accuracy of IOP measurement and the detection IOP changes should not be overlooked. Sung et al [20] investigated the clinical characteristics of glaucomatous subjects who underwent LRS. They reported similar levels of IOP reduction after using anti-glaucoma medication between glaucomatous eyes with a history of LRS and glaucomatous eyes without such a history. Their study indicated the possible validity of using GAT for detecting IOP changes in glaucoma patients who have undergone LRS. GAT is considered to be the gold standard for IOP measurement and is commonly used in the glaucoma field. Therefore, if IOP changes can be monitored using GAT, glaucoma patients with the history of LRS can receive timely examination and/or treatment for the condition.

In the present study, OAG patients who underwent LASIK or LASEK were divided into one of two subgroups according to GAT fluctuation. By definition, IOP fluctuation indicates the degree of IOP change during the follow-up period. Therefore, if IOP fluctuation is high, IOP changes must be monitored carefully because there is possibility of glaucoma progression. In addition, GAT has been generally used in glaucoma clinics because it is regarded as the gold standard for IOP measurement. For such reasons, we decided to divide our patients into groups according to GAT fluctuation values. By using GAT fluctuation as a standard and comparing with IOP fluctuation using DCT, which is known for relatively accurate IOP measurement method for patients with a history of laser refractive surgery, the value of IOP monitoring using GAT can be verified. Mean and peak IOP measured using DCT were significantly higher than those measured using GAT, regardless of study group. These results correspond with previous studies that compared IOP values in patients who underwent LASIK or LASEK and various tonometry methods [9, 12, 13]. Interestingly, although the mean values of IOP parameters for IOP change (i.e., IOP fluctuation and IOP reduction) were higher in the HIFG than the LIFG, and mean ΔIOP fluctuation was significantly smaller in the HIFG. Considering the Bland-Altman plot of GAT and DCT fluctuation (Fig 1), agreement between these two fluctuation values were high. In correlation analyses, there was no significant correlation between DCT and GAT fluctuation in the LIFG; however, there was significant correlation in the HIFG. Despite adjusting for other factors, such as age, sex, operation type, ocular parameters, and mean IOP, correlation between GAT fluctuation and DCT fluctuation was maintained in the multivariate analyses. Overall, IOP fluctuation in OAG patients who underwent LRS exhibited similar values between two different tonometries, and the difference became smaller when the IOP fluctuation measured using GAT was relatively high. IOP monitoring is important for glaucoma patients who exhibit a large range of IOP change. When we monitor IOP change in glaucoma patients who have undergone LRS, the best is to use DCT. However, there is a high possibility that DCT is not readily available in typical real-world clinical situations. In the present study, we set IOP fluctuation using GAT as a standard for dividing the groups to reflect clinical situation(s). After that, we compared GAT fluctuation with DCT fluctuation, and verified that these two fluctuation vales were similar and differences were decreased as the GAT fluctuation was increased. This result supports the validity of IOP monitoring using GAT in OAG patient with a history of LRS.

In assessing IOP, there are several factors that should be considered. CCT is a well-known factor related to IOP. In normal eyes, IOPs measured using GAT are higher in individuals with thicker corneas and lower in thinner corneas. However, in glaucomatous eye(s), this correlation is variable. Although it is widely known that IOP measurement using DCT is not significantly influenced by CCT compared with GAT, previous studies have reported that both GAT and DCT measurement values demonstrate significant correlation with CCT [21, 22] or non-significant correlation with CCT in glaucomatous eyes [23, 24]. CC is also affected by IOP measurement method. There have been varying correlation results between IOP values and CC depending on whether the study investigating altered CC involved normal eyes or pathologic eyes [21, 25–29]. CCT and CC are altered by LRS. Therefore, in glaucomatous eyes with a history of LRS, investigating the correlation between IOP parameters and CC, or CCT according to tonometry method, is important. Although AXL is considered to be a correlated factor in IOP measurement, several studies have reported no significant correlations [27, 30, 31]. Nevertheless, Loewen et al [32] reported that AXL had a significantly negative correlation with 24 h IOP fluctuation. In our univariate analyses, CCT, CC, and AXL demonstrated significant correlation with GAT fluctuation in the HIFG, and AXL showed significant correlation with DCT fluctuation in the LIFG. Although these correlations disappeared in the multivariate analyses, our results suggest that some factors, including CCT, CC, and AXL, may have different correlations with IOP in glaucoma patients who have undergone LRS, unlike the results of previous studies. Therefore, further studies are needed to evaluate the exact correlation among these factors in glaucoma patients with the history of LRS. In addition, we should consider the effects of CCT, CC and AXL on IOP monitoring in these patients.

One particular limitation of the present study was its retrospective design; however, we had no choice but to rely on the veracity of medical records. To overcome the inherent limitations associated with retrospective study designs, we applied strict prerequisites when collecting data. Consequently, the number of subjects included was not large. In addition, if there were other subjects that could have served as a control group (for example, OAG patients without a history of LRS under the same conditions, or normal subjects with a history of LRS), additional analyses for the GAT value of monitoring IOP change in OAG patients with a history of LRS could be conducted.

## Conclusions

In the present study, IOP parameters calculated using GAT and DCT demonstrated significant correlation in OAG patients who underwent LRS, although their actual value(s) was different. In the group consisting of patients with high GAT fluctuation, significant correlation between GAT fluctuation and DCT fluctuation was still verified in the multivariate analysis. IOP change is important when we monitor glaucoma patients. However, there is no established consensus as to which mode of tonometry is the most accurate for monitoring IOP in OAG patients who have undergone LRS, although DCT is regarded to be a relatively exact method for IOP measurement in this patient group. By demonstrating significant correlation between DCT fluctuation and GAT fluctuation in HIFG, our results suggest that it is acceptable to apply GAT for monitoring IOP in OAG patients with a history of LRS. However, because GAT fluctuation was correlated with CCT, AXL and CC in HIFG, these factors should be considered when using GAT for monitoring.

## Supporting information

S1 Dataset Mininal data set. (PDF)
