# Correction: Utility of Goldmann applanation tonometry for monitoring intraocular pressure in glaucoma patients with a history of laser refractory surgery

**DOI:** 10.1371/journal.pone.0206564

**Published:** 2018-10-25

**Authors:** Sang Yeop Lee, Hyoung Won Bae, Hee Jung Kwon, Gong Je Seong, Chan Yun Kim

In the title of the article, the word “refractory” should be “refractive.” The correct title is: Utility of Goldmann applanation tonometry for monitoring intraocular pressure in glaucoma patients with a history of laser refractive surgery. The correct citation is: Lee SY, Bae HW, Kwon HJ, Seong GJ, Kim CY (2018) Utility of Goldmann applanation tonometry for monitoring intraocular pressure in glaucoma patients with a history of laser refractive surgery. PLoS ONE 13(2): e0192344. https://doi.org/10.1371/journal.pone.0192344
