# Supplementary material for: Utility of Goldmann applanation tonometry for monitoring intraocular pressure in glaucoma patients with a history of laser refractory surgery
Source: PLoS One. 2018 Feb 5;13(2):e0192344. doi: 10.1371/journal.pone.0192344 (PMC5798809; doi:10.1371/journal.pone.0192344)
Supplement: S1 Dataset — (PDF) [file pone.0192344.s001.pdf]

OD/OS	Age	Sex	F/U duration	Type of refractive surgery (1=LASIK, 2=LASEK, 3=EXCIMER)	Year of op
1	41	2	34	2	2003
2	41	2	34	2	2003
1	48	1	23	1	1999
2	48	1	23	1	1999
1	46	2	15	1	2011
2	46	2	15	1	2011
2	41	2	17	1	2003
1	40	1	28	2	2012
1	35	1	52	2	2001
2	35	1	52	2	2002
1	55	2	85	1	2000
2	55	2	85	1	2000
1	34	1	64	1	2003
2	34	1	64	1	2003
1	41	1	56	3	1995
1	43	2	48	1	2010
2	43	2	48	1	2010
1	38	1	50	1	2005
2	38	1	50	1	2005
1	39	2	45	2	2005
2	39	2	45	2	2005
1	49	2	20	1	2003
2	49	2	20	1	2003
1	57	1	38	1	2002
2	57	1	38	1	2002
1	37	1	33	2	2012
2	37	1	33	2	2012
1	44	2	30	3	1992
2	44	2	30	3	1992
1	30	2	28	1	2004
2	30	2	28	1	2004
1	34	2	25	2	2005
2	34	2	25	2	2005
1	31	1	19	2	2007
2	31	1	19	2	2007
1	30	2	21	2	2009
2	30	2	21	2	2009
1	50	1	23	2	2006
2	50	1	23	2	2006

1	46	2	23	2	2003
2	46	2	23	2	2003
1	44	2	24	2	2006
2	44	2	24	2	2006
1	41	1	16	1	2000
2	41	1	16	1	2000
2	31	2	15	2	2005
1	33	1	15	2	2010
2	33	1	15	2	2010
1	37	2	106	2	2006
2	37	2	106	2	2006
1	45	1	114	3	1995
2	45	1	114	1	1996
1	42	2	19	1	1997
2	42	2	19	1	1998
1	36	2	4	1	2005
2	36	2	4	1	2005
1	55	1	222	1	1999
2	55	1	222	1	1999
1	42	2	18	1	2002
2	42	2	18	1	2002
1	36	2	104	2	2006
2	36	2	104	2	2006
2	40	1	28	2	2012
1	43	1	30	2	2012
2	43	1	30	2	2012
1	39	2	29	1	2000
2	39	2	29	1	2000
1	36	2	187	1	2005
2	36	2	187	1	2005
1	57	2	48	3	1997
2	37	2	48	3	1997
1	35	2	37	2	2006
2	35	2	37	2	2006
1	37	2	68	1	2000
2	37	2	68	1	2000
1	45	1	81	1	2000
2	45	1	81	1	2000
1	43	1	85	1	2001
2	43	1	85	1	2001
1	47	1	79	1	2000
2	47	1	79	1	2000
1	53	2	80	1	2001

1	43	1	71	1	2003
2	43	1	71	1	2003
2	33	1	46	1	2002
1	42	2	50	1	2001
2	42	2	50	1	2001
1	43	1	48	1	1996
2	43	1	48	1	1996
1	49	2	47	1	2002
2	49	2	47	1	2002
1	27	2	42	2	2009
1	30	2	24	1	2012
2	30	2	24	1	2012
1	36	2	104	2	2006
2	36	2	104	2	2006
1	32	2	16	1	2002
2	32	2	16	1	2002
1	62	2	48	1	2000
2	62	2	48	1	2000
1	64	1	53	3	1990
2	64	1	53	3	1990
1	31	2	29	2	2011
2	31	2	29	2	2011
1	29	1	30	2	2012
2	29	1	30	2	2012
1	51	1	105	2	2004
2	51	1	105	2	2004
1	33	1	46	1	2002
1	42	1	4	3	1995
2	42	1	4	3	1995
1	42	1	90	1	2000
2	42	1	90	1	2000
1	27	2	60	1	2008
2	27	2	60	1	2008
1	48	2	77	1	2001
2	48	2	77	1	2001
1	56	2	72	1	2004
2	56	2	72	1	2004
2	41	1	56	3	1995
1	40	2	31	1	2002
2	40	2	31	1	2002
1	45	2	42	1	2001
2	45	2	42	1	2001
1	37	1	53	1	2009

2	37	1	53	1	2009
1	55	2	47	2	2005
2	55	2	47	2	2005
1	35	2	43	2	2009
2	35	2	43	2	2009
1	36	2	18	1	2003
2	39	2	18	1	2003
1	32	2	22	1	2010
2	32	2	22	1	2010
1	31	2	35	1	2006
2	31	2	35	1	2006
1	30	2	36	1	2006
2	30	2	36	1	2006
1	40	2	17	1	1999
2	40	2	17	1	1999
1	31	2	15	2	2005

Duration (Y)	CCT	AXL	SE	LAST BCVA	CC	medicaitno
12	424		-1.75	1		1
12	402		-1.5	1		1
16	480	27.57	-2.5	1	38.48	1
16	479	27.23	-1.75	1	38.45	1
4	502	25	-0.88	1	43.19	1
4	482	24.67	0.5	1	42.6	1
12			-2.5	0.8	39.26	0
3	455	26.55	-1.5	1	39.25	1
14	423	28.23	-1.87	1	35.03	0
13	418	28.06	-0.5	1	33.88	0
15	518	28.24	-3.75	1	39.95	1
15	446	28.81	-1.75	1	37.69	1
12	440	26.98	-1.37	1	36.11	1
12	454	26.79	-0.12	1	36.27	1
20	498		-0.25	1		1
5	508		0.25	1		1
5	622		-0.25	1		1
10	529	26.43	-1.75	1	37.83	1
10	521	26.42	-1.5	1	37.78	1
10	431	26.49	-0.5	1	38.16	1
10	430	26.5	-0.25	1	38.03	1
12	496		-1.5	1		1
12	501		-3	0.8		1
13	492		0.75	0.8		1
13	494		-0.25	1		1
3	372	28.01	-0.25	1	35.23	1
3	385	28	-0.25	1	34.99	1
23	477		-2.75	0.9		1
23	484		-2.5	0.9		1
11	429	25.9	-1.75	1	38.8	1
11	428	25.64	-1	1	39.28	1
10	409	27.99	-1.12	1	35.08	1
10	407	28.02	-1	1	34.89	1
8	465	27.26	-1.5	1	36.27	1
8	459	27.22	-1.25	1	36.07	1
6	452		-1.25	1		1
6	462		-1.5	1		1
9	526	24.96	-1	1	41.53	1
9	529	25.01	-1.75	1	41.5	1

12	497	27.23	-1.37	1	39.63	1
12	501	26.89	-1.25	1	39.84	1
9	440	25.85	-1.5	1	39.09	1
9	443	25.8	-1.5	1	39.25	1
15	463	28.67	-3	0.9	35.99	0
15	445	28.62	-2.75	0.9	36.18	0
10	492		-2.25	1		1
5	429		-0.5	1		1
5	430		0	1		1
9	457	25.32	-0.5	1	39.32	0
9	458	25.44	0.125	1	39.23	0
20	377	27.67	-1.625	0.9	36.38	1
19	388	27.12	-4	0.9	38.95	1
18	389	33.66	-10.25	0.5	35.28	1
17	400	33.53	-11.5	0.3	36.16	1
10	482	28.65	-3.25	1	36.23	1
10	479	28.64	-2	1	35.63	1
16	504	26.43	-1.5	1	38.09	1
16	497	27.33	-1	1	36.58	1
13	529	24.62	-1.25	1	42.81	1
13	520	24.19	-0.25	1	42.61	1
9	391	27.84	-3.5	1	36.37	1
9	393	27.44	-2.75	1	36.16	1
3	455	26.63	-1	1	39.25	1
3	436	28.97	-1.75	1	36.24	1
3	439	28.96	-1.87	1	36.42	1
15	509	25.32	-2	1	38.7	1
15	493	26.12	-1	1	36.79	1
10		24.86	0.37	1	40.06	1
10		25.14	0.87	1	39.52	1
18	488	25	0.495	1	41.19	1
18	489	25.51	1.185	0.8	40.79	1
9	424	26.96		1	37.11	1
9	466	25.68		1	40.06	1
15	499	27.5	-3.75	1	37.65	1
15	487	27.9	-2	1	36.95	1
15	445	25.11	0.75	1	38.47	1
15	445	24.91	0.25	1	39.58	1
14	478	29.17	-2.75	1	35.19	1
14	480	29.4	-2.75	1	38.29	1
15	475	26.05	-1	1	40.97	1
15	470	26.14	-1	1	40.23	1
14	556	24.64	-0.5	1	41.65	1

12	546	25.47	-0.75	1	39.84	1
12	546	25.21	-0.62	1	40.39	1
13	444	30.54	-3	0.6	30.23	1
14	486	25.63	-1.25	1	40.5	1
14	488	25.59	-1.25	1	41.26	1
19	551	27.66	-3.75	0.8	36.79	1
19	529	27.81	-3.25	0.8	36.3	1
13	447	27.51	-3	0.5	36.16	1
13	450	27.41	-1.75	0.5	35.27	1
6	378	28.03	-1.5	0.3	35.47	1
3	463		-0.25	1		1
3	449		0.25	1		1
9	391	27.84	-3.5	1	36.37	1
9	393	27.44	-2.75	1	36.16	1
13	522		-1.5	1		1
13	512		-0.5	1		0
15	445	31.36	-5.87	0.8		1
15	471	26.79	-3.37	0.7		1
25	421	25.88	-2.25	0.5		1
25	419	25.95	-4.5	0.9		1
4	402	25.35				1
4	390	25.41				1
3	415	26.67	-0.5	1		0
3	431	26.28	-0.5	1		1
11	473	27.5	-2.75	0.2		1
11	449	27.77	-0.75	0.9		0
13	421	30.64	-4.75	0.3		1
20	474	27.21	-3	1		1
20	483	27.56	-3.25	1		1
15	474	27.69		1		0
15	469	27.41		1		0
7	438	24.9	-0.37	1		0
7	424	25.27	-0.5	1		0
14	498	25.07	-0.5	1		0
14	500	24.83	-0.5	1		0
11	430	27.53	-1.25	1		0
11	480	25.89	-0.87	1		0
20	497		-0.5	1		0
13	485		-0.37	1		1
13	480		-0.37	1		1
14	457	25.45	-0.37	1		0
14	446	25.9	-0.25	1		0
6	506	25.76	0	1		0

6	492	25.67	0.25	1	0
10	484	25.5	-1	0.9	1
10	487	25.59	-0.5	1	1
6	513	25.84	0.25	1	0
6	507	26.35	-0.25	1	0
12		24.88	0.25	1	0
12		24.96	0.25	1	0
5	469	27.01	-0.5	1	0
5	465	26.76	-0.75	1	0
9	425	27.23	-1	1	0
9	425	27.29	-1.25	1	0
9	448		-1.5	1	0
9	479		0.75	1	0
16	520	25.08	-0.5	1	0
16	514	25.13	-1	1	0
10	462		-1.75	1	0

Medication change 0= no 1=stable, 2 change	Number of change	Number of change /mon	GAT mean	GAT peak	fluctuation	AT reduction
1	0	0	12.000	15.000	2.160	3.000
1	0	0	11.857	15.000	2.340	3.143
1	0	0	10.000	11.000	1.732	1.000
1	0	0	11.000	13.000	2.646	2.000
1	0	0	12.000	14.000	1.155	2.000
1	0	0	11.714	14.000	1.604	2.286
1	0	0	12.250	13.000	0.957	0.750
1	0	0	13.111	18.000	1.965	4.889
1	0	0	11.800	13.000	1.643	1.200
1	0	0	12.000	13.000	1.414	1.000
1	0	0	13.714	15.000	0.951	1.286
1	0	0	12.571	15.000	1.618	2.429
1	0	0	10.143	14.000	2.911	3.857
1	0	0	10.429	13.000	2.760	2.571
1	0	0	12.667	15.000	2.082	2.333
1	0	0	13.250	16.000	1.893	2.750
1	0	0	13.500	16.000	1.915	2.500
1	0	0	9.714	11.000	0.951	1.286
1	0	0	9.857	11.000	1.069	1.143
1	0	0	12.333	14.000	1.033	1.667
1	0	0	12.333	14.000	1.033	1.667
1	0	0	13.500	15.000	1.291	1.500
1	0	0	13.250	15.000	1.258	1.750
1	0	0	13.667	22.000	3.464	8.333
1	0	0	14.333	22.000	2.958	7.667
1	0	0	7.333	8.000	0.577	0.667
1	0	0	8.333	9.000	0.577	0.667
1	0	0	10.000	12.000	1.414	2.000
1	0	0	12.400	13.000	0.894	0.600
1	0	0	8.333	13.000	2.422	4.667
1	0	0	8.667	11.000	1.211	2.333
1	0	0	11.500	13.000	2.380	1.500
1	0	0	13.250	17.000	2.872	3.750
1	0	0	8.000	12.000	2.345	4.000
1	0	0	9.200	10.000	1.095	0.800
1	0	0	8.667	10.000	1.033	1.333
1	0	0	9.000	11.000	1.414	2.000
1	0	0	12.333	14.000	1.366	1.667
1	0	0	12.167	14.000	1.722	1.833

1	0	0	12.500	14.000	1.915	1.500
1	0	0	14.500	16.000	1.915	1.500
1	0	0	7.000	7.000	0.000	0.000
1	0	0	7.333	8.000	0.577	0.667
1	0	0	10.750	12.000	1.258	1.250
1	0	0	10.750	13.000	1.708	2.250
1	0	0	12.833	15.000	1.722	2.167
1	0	0	7.500	9.000	1.732	1.500
1	0	0	6.750	9.000	2.062	2.250
2	2	0.226415	13.250	15.000	1.500	1.750
2	2	0.226415	11.250	14.000	1.893	2.750
2	2	0.210526	8.000	11.000	1.673	3.000
2	2	0.210526	6.333	7.000	0.816	0.667
2	1	0.631579	12.250	15.000	2.754	2.750
2	1	0.631579	11.250	15.000	2.630	3.750
2	1	3	12.333	15.000	2.517	2.667
2	1	3	16.000	17.000	1.732	1.000
2	3	0.162162	11.000	12.000	1.000	1.000
2	3	0.162162	11.000	12.000	0.707	1.000
2	4	2.666667	10.429	13.000	1.718	2.571
2	4	2.666667	10.429	12.000	1.397	1.571
2	9	1.038462	10.000	15.000	2.483	5.000
2	9	1.038462	12.077	16.000	2.431	3.923
2	1	0.428571	13.222	18.000	2.386	4.778
2	2	0.8	16.556	20.000	2.877	3.444
2	2	0.8	12.222	14.000	1.481	1.778
2	4	1.655172	12.000	16.000	2.268	4.000
2	4	1.655172	12.375	17.000	2.066	4.625
2	2	0.128342	10.500	13.000	1.716	2.500
2	2	0.128342	11.100	12.000	0.876	0.900
2	1	0.25	10.750	12.000	1.035	1.250
2	2	0.5	11.500	13.000	1.604	1.500
2	2	0.648649	11.333	15.000	2.066	3.667
2	2	0.648649	12.167	16.000	2.483	3.833
2	2	0.352941	7.200	10.000	1.924	2.800
2	2	0.352941	6.800	8.000	0.837	1.200
2	1	0.148148	10.750	17.000	2.866	6.250
2	1	0.148148	10.625	14.000	1.847	3.375
2	2	0.282353	10.857	14.000	1.748	3.143
2	2	0.282353	11.571	14.000	1.453	2.429
2	2	0.303797	8.500	13.000	2.593	4.500
2	2	0.303797	9.600	15.000	2.459	5.400
2	2	0.3	13.182	16.000	1.991	2.818

2	1	0.169014	11.222	15.000	1.787	3.778
2	1	0.169014	11.667	14.000	1.658	2.333
2	3	0.782609	8.000	10.000	1.414	2.000
2	2	0.48	11.364	13.000	1.027	1.636
2	2	0.48	11.273	13.000	1.794	1.727
2	3	0.75	12.000	17.000	2.138	5.000
2	3	0.75	11.250	17.000	2.493	5.750
2	1	0.255319	10.571	13.000	1.718	2.429
2	1	0.255319	11.571	15.000	2.507	3.429
2	4	1.142857	14.455	22.000	3.320	7.545
2	2	1	11.444	13.000	1.130	1.556
2	2	1	11.556	13.000	1.333	1.444
2	7	0.807692	10.267	15.000	2.434	4.733
2	7	0.807692	12.467	16.000	2.264	3.533
2	1	0.75	11.200	13.000	2.168	1.800
2	1	0.75	12.200	14.000	1.483	1.800
2	2	4.166667	13.250	17.000	3.304	
2	2	4.166667	14.250	17.000	2.500	
2	1	1.886792	8.667	10.000	0.816	
2	2	3.773585	8.667	11.000	2.582	
2	3	10.34483	10.389	19.000	3.648	
2	4	13.7931	11.833	17.000	3.092	
2	1	3.333333	10.778	15.000	2.587	
2	2	6.666667	10.889	16.000	4.343	
2	5	4.761905	11.811	36.000	8.965	
2	5	4.761905	11.914	30.000	5.782	
1	2	4.347826	5.200	6.000	0.837	
2	2	50	10.250	12.000	1.500	
2	2	50	12.750	16.000	2.217	
0	0	0	11.200	13.000	1.304	
0	0	0	11.400	13.000	1.140	
0	0	0	12.800	16.000	2.490	
0	0	0	11.800	13.000	1.643	
0	0	0	12.250	14.000	2.062	
0	0	0	11.250	13.000	2.363	
0	0	0	9.500	11.000	2.121	
0	0	0	11.000	12.000	1.414	
0	0	0	12.667	15.000	2.082	
0	0	0	10.667	12.000	1.033	
0	0	0	10.667	12.000	1.033	
0	0	0	12.250	13.000	0.957	
0	0	0	11.750	12.000	0.500	
0	0	0	12.500	13.000	0.707	

	0	0	0	12.000	13.000	1.414
	0	0	0	13.250	17.000	2.872
	0	0	0	14.000	19.000	3.559
	0	0	0	11.000	14.000	2.000
	0	0	0	11.250	15.000	2.500
	0	0	0	12.000	12.000	0.000
	0	0	0	12.000	12.000	0.000
	0	0	0	10.667	12.000	1.155
	0	0	0	11.000	13.000	1.732
	0	0	0	11.750	14.000	1.708
	0	0	0	11.750	13.000	1.500
	0	0	0	10.500	17.000	3.391
	0	0	0	10.000	12.000	1.414
	0	0	0	14.250	16.000	1.708
	0	0	0	15.500	17.000	1.732
	0	0	0	14.000	16.000	2.280
